# Disasters, Diagnosis, and Distress: Multiple Perspectives, Populations, and Methodologies

**DOI:** 10.3390/bs12050152

**Published:** 2022-05-18

**Authors:** Carol S. North, David E. Pollio, Elizabeth Whitney Pollio

**Affiliations:** 1 The Altshuler Center for Education and Research, Metrocare Services, Dallas, TX 75247, USA; 2 Department of Psychiatry, University of Texas Southwestern Medical Center, Dallas, TX 75390, USA; 3 Private Practice, Birmingham, AL 35294, USA; davpoll62@yahoo.com; 4 UAB Center for Study of Community Health, School of Public Health, University of Alabama at Birmingham, Birmingham, AL 35294, USA; ewpollio@uab.edu; 5 School of Nursing, University of Alabama at Birmingham, Birmingham, AL 35294, USA

## 1. Introduction

Disaster mental health is a consequential topic in today’s world in which disasters are increasing in both numbers and magnitude and inflicting deep psychological wounds across wide populations. The succession of major disasters of many types in the last several decades has been followed by a rapid accumulation of disaster mental health literature examining post-disaster psychosocial outcomes. Ongoing research addressing evolving characteristics of disasters across the globe has exposed the limitations of traditional ways of conducting disaster research, necessitating development of innovative approaches for previously uncontemplated research challenges. Research has increasingly revealed that disasters lead to many different types of mental health sequelae including nonpathological distress as well as PTSD and other psychiatric disorders, all of which require different approaches directed by an accurate assessment of post-disaster difficulties. Continued new research responding to the evolving characteristics of disasters and their varied mental health sequelae is vital to helping guide the best mental health responses for the previously unimaginable circumstances presented by current and future disasters.

This Special Issue tackles these varied and evolving research challenges in a collection of articles addressing indispensable as well as underappreciated aspects of research on mental health effects of disaster and mass trauma in methodologically differing studies in adults and children, including the special case of the recent COVID-19 pandemic. The studies in this collection cover events across the full disaster typology (natural disasters, technological accidents, and intentional human-caused incidents including terrorism). Some of these studies recruited directly exposed disaster trauma survivors, but others examined other disaster-affected populations. An array of research methods and designs found in these studies includes the use of different assessment tools (e.g., symptom self-report questionnaires vs. structured diagnostic interviews), quantitative and qualitative data approaches, epidemiologic/descriptive designs and intervention studies, and different times of assessment from early (from weeks to months) to longer-term (from years to decades) post-disaster periods and even pre-disaster time frames. The authors of the articles in this Special Issue bring a broad array of internationally recognized expertise in psychiatric nosology and research methodology arising from their extensive, longstanding, and pioneering disaster mental health research experience. In these articles, they have contributed extraordinary depth and rigor to unique angles of investigation from several perspectives, providing illumination of many critically important facets of this research.

## 2. Selected Articles on Disaster and Mass Trauma Mental Health Research with Adult and Child Populations

Of the six original research articles and two review articles on disaster and mass trauma mental health included in this Special Issue, four articles focused on adult populations. A problem with the many differences in various aspects of the research methods across the included studies is that it makes a direct comparison of the findings difficult. However, a benefit of this variability is that it invites discussions on how the different methods used and the various aspects of mental health examined in this literature relate to the findings.

### 2.1. Disaster Mental Health Research in Adults

The study by Tucker et al. [1] in this Special Issue examined a random sample of 138 directly exposed Oklahoma City bombing survivors almost 19 years after the bombing and compared them with 171 unexposed community members. The investigation of psychosocial adjustment of disaster survivors after the passage of decades in this study provides much longer-term information than is available in most disaster studies; however, a lack of data collected earlier in the post-disaster course of this sample introduces potential for corruption or loss of recollection over such a long period.

The quantitative data collected using symptom checklists in the Tucker et al. study revealed that exposure to the bomb blast was associated with posttraumatic stress, depression, and anxiety but not health problems or medical or mental health care in the last year. Additionally, extensive evidence of posttraumatic growth was reported by about one-third of the survivors in this study, knowledge obtained because the collection of data was not limited to psychopathology and other negative responses to disaster experience.

An interesting part of the Tucker et al. study was the inclusion of qualitative data collected through open-ended questions that complemented, extended, and expanded the quantitative data obtained through the structured limited-response questions, allowing for a comparison of constructs across both quantitative and qualitative data collected in this single study. Besides confirming the posttraumatic and other psychiatric symptoms and problems related to injuries and health effects identified in the quantitative data, the qualitative data revealed personal problems and intervention needs related to work, education, finances, housing, and interpersonal matters. Additionally, the qualitative data uniquely generated poignant expressions of the survivors’ bombing-related problems in descriptions not provided in responses to the structured quantitative queries. This article concluded that data collected from qualitative methods may differ from quantitative data in intensity and subjective description of experience and that reliance on quantitative research questionnaires may miss many issues that survivors may voice in reporting their personal experience.

Two original research articles in this Special Issue by North and Baron [2,3] examined major depressive disorder (MDD) in studies of survivors of 11 different disasters representing the range of disaster typologies. The application of consistent research methods (examining survivors directly exposed to disaster trauma, collection of data within a few months of the disaster, and use of structured diagnostic interviews assessing pre-disaster and post-disaster psychiatric disorders) allowed the researchers to assemble the data from 10 of these disaster studies into a single large database of 1181 survivors. This is rarely possible in disaster research studies, as important methodological differences hinder or prohibit comparison or combination of databases. The similarities between these research methods in a study conducted by the same research group on the 11 September 2001 (9/11) attacks on the World Trade Center in New York City further allowed for a comparison and contrast of the combined 10-disaster sample with the 9/11 sample and replication of the analyses across the combined 10-disaster and 9/11 disaster datasets. A pivotal methodological advantage of all of these studies was the careful attention and adherence to the definition of trauma exposure and its distinction from other stressors, instilling confidence that the findings actually represent disaster trauma outcomes.

The first of these two studies [2] examined the symptom structure of post-disaster MDD, finding consistent symptom patterns defining MDD in both samples and no specific symptom clusters identifying MDD, which was characterized by a cohesive grouping of all of its symptoms. The main implication of these findings was that the evidence did not suggest the potential for development of brief symptom screeners for post-disaster MDD, so that a full diagnostic assessment for the disorder remains necessary. The second of these two studies [3] found MDD in the 9/11 data to be the most common post-disaster disorder and twice as prevalent as in the combined dataset of other disasters in which disaster-related PTSD was the most prevalent disorder. Additionally, MDD remission status was associated with employment and marital status but not with overall functioning, generating potential avenues for exploration of directions for disaster-response interventions addressing post-disaster psychosocial adjustment.

An original research article in this Special Issue by Pfefferbaum et al. [4] examined MDD in relation to 9/11 news media consumption in a subset of 254 members of the same 9/11 dataset collected by the team of North and colleagues included in the two studies above. The methodological advantages of this dataset include not only structured diagnostic interviews but also the separate analysis of MDD diagnosis, symptoms, and associated levels of functioning. Additionally, incident (new) MDD and depressive symptoms after the disaster were specifically examined separately from pre-disaster and post-disaster prevalence. This allowed the analysis to focus on incident (new) psychiatric disorders and symptoms that are most likely to be disaster-related (unlike *all* postdisaster disorders and symptoms, many or most of which may be unrelated to the disaster, as indicated by their pre-disaster origins). Another strength of this study’s methods was that it compared the findings with different disaster trauma exposure groups (direct, witnessed, and indirect exposure via close associates and non-exposure).

These detailed methodological provisions allowed for the determination that post-9/11 news contact was associated with post-disaster persistent/recurrent and incident depressive symptoms in unexposed and indirectly exposed groups but not in those directly exposed to 9/11 disaster trauma possibly because the intense disaster experience of directly exposed individuals may have outweighed the effects of news media contact. These findings provide evidence suggesting that clinical and public health attention to contributions of media consumption to post-disaster depression focus primarily on groups without direct disaster trauma exposure.

### 2.2. Disaster Mental Health Research in Children

Two articles in this Special Issue were focused on child disaster mental health, one an original research study and the other a meta-analytic review. Children have been far less studied than adult populations in disaster mental health research. A separate study of children is warranted as their mental health outcomes and needs may differ from those of adults and as different approaches to disaster mental health interventions may be required.

The original research study by Lee et al. [5] investigated the mental health effects of disaster on children and their families exposed to dioxin contamination in Times Beach, MO and the flooding in the St. Louis, MO area in 1982. This article provided an important lesson in the value of older data. The dataset for this NIMH-funded study was collected in 1986–87, but before the study could be published, the principal investigator and her mentor had both passed away. This vintage dataset was resurrected by their mentee, and without funding to support the processing and analysis of the existing data and writing the results into an article, the original researchers’ mentee (senior author of this article) succeeded in completing the published article from this study three and a half decades after the data were originally collected. This work also provided a research training opportunity for four mentees of this article’s senior author who are coauthors (including the first author) of the published article. The age of the data was not without complaints from reviewers. Respected senior experts in scientific methodology [6] have, however, cautioned that older data are not necessarily less valid or of lower quality because of their age and that research should be judged and valued based on the rigor of the study rather than dismissed simply because of its age.

A testament to the wisdom of this journal’s editorship is that it recognized the value of this older study and published that article. The Lee et al. study and its vintage dataset exhibit unparalleled methodological rigor with structured diagnostic interviews of a household sample of 290 children as well as their parents (and other family members) within 169 families selected randomly from the disaster-stricken areas, achieving 87% participation of eligible families. This study was unique in that no prior studies have conducted structured diagnostic interviews (rather than symptom scales, nondiagnostic constructs, or nonsystematic clinical diagnoses) of both parents and their children in such a large disaster-exposed sample (total *n* = 562). Furthermore, the study was not limited to examination of posttraumatic stress disorder (PTSD) and included assessments of a total of 11 psychiatric diagnoses (including also depressive, anxious, behavioral, and substance use disorders). Additionally, this study assessed not only pre-disaster (retrospectively) and post-disaster disorders but also incident (new) psychiatric disorders representing the psychopathology that is most likely to be disaster-related.

The Lee et al. study found that disaster-related disorders including PTSD in children were highly associated with those of their parents, especially their mothers. Because the mental health outcomes of children in disaster may be intertwined with those of their parents, it was suggested that clinicians should conduct interventions with parents and children together. Therefore, it can be seen that this vintage study was uniquely valuable in its large and representative disaster sample with diagnostic interview data in both children and their parents, and in other adult family members, allowing for new insights by comparing carefully assessed child and parent disaster-related psychopathology. Had this article been summarily dismissed based on the age of the data alone, valuable new information would have been lost and the government funding of this major study would essentially have been wasted.

A review article by Pfefferbaum et al. [7] in this Special Issue synthesized the results of 12 meta-analyses of randomized controlled trials of outcomes (PTSD, depression, anxiety, and functional impairment) of interventions for mass trauma in children. The mass trauma events studied included a broad array of incidents, including natural disasters, human-made disasters (technological accidents, mass violence, and armed conflict), and traumatic events in humanitarian settings in low- and middle-income countries as well as incidents in high-income settings. The types of interventions studied in the articles reviewed were psychological treatment, non-pharmacologic psychological and behavior interventions, focused psychosocial interventions involving emotional and practical support, and mental health or psychosocial support practice.

Altogether, the studies reviewed in the Pfefferbaum et al. article demonstrated small-to-moderate benefit for PTSD and small-to-nonsignificant benefit for depression, anxiety, and functional impairment. None of the outcomes differed across different types of mass trauma. Demographic factors and disaster trauma exposure levels did not appear to relate to intervention outcomes. A key finding from a subgroup analysis of the studies was that interventions can be beneficial for PTSD outcomes of children exposed to mass trauma. The main take-home message for clinical practice implications was that decisions about the choice and administration of interventions need to be matched to the populations being served and that the context of the situation and specific interventions must be targeted for specific psychological issues. An important caveat was the recognition that many children’s trauma reactions represent normal adjustment to extreme events and circumstances rather than psychopathology, thus warranting not formal treatment for psychopathology but disaster mental health response interventions addressing psychosocial adjustment.

## 3. Selected Articles on the COVID-19 Pandemic

Infectious disease pandemics have plagued humankind for thousands of years, killed millions to billions of people, eradicated large portions of entire populations, and altered pivotal junctures in the course of world history. Notorious pandemics in history include the Middle Eastern bubonic plague (Yersinia pestis carried by fleas) beginning in the Middle Ages and spreading through Africa, Asia, and Europe and continuing into the 1600s as Black Death, which is considered the most fatal pandemic ever recorded in human history. A more recent pandemic considered the most severe pandemic in recent history was the 1918 so-called “Spanish flu” global pandemic with unknown origins (H1N1 influenza A virus). Other notable recent epidemics include the Severe Acute Respiratory Syndrome (SARS) outbreak emerging from China in 2002 and spreading to four continents, the 2009 H1N1 swine flu pandemic originating in Mexico and disseminating worldwide, and the Ebola outbreak beginning in West Africa in 2014 and infiltrating other continents. The novel coronavirus disease 2019 (COVID-19), caused by severe acute respiratory syndrome coronavirus 2 (SARS-CoV-2), emerged in 2019 in Wuhan, China, and rapidly spread throughout the world to become a currently ongoing crisis rated as one of the deadliest pandemics in human history.

Pandemics represent a type of natural disaster. Naturally occurring illnesses of pandemics cause widespread death, medical morbidity, suffering, and far-reaching social and economic consequences. Published research especially since the SARS outbreak of 2002 has increasingly investigated the mental health effects of these disasters. The psychosocial effects on populations with COVID-19 have been shown to be far-reaching, including not only people infected with COVID-19 but also their family and friends, medical professionals and healthcare systems, and members of general populations through fears of being infected, social isolation, and economic devastation. Research articles investigating the psychosocial effects of the COVID-19 pandemic rapidly entered the literature in early 2020, with accelerating proliferation thereafter.

Disaster mental health research in general has focused on PTSD far more than on other psychopathology; PTSD has been considered the “signature diagnosis of disaster” [8]. Natural disasters involving pandemics seem to be following this direction. A perspective article in this Special Issue by North et al. [9] provides a nosological exploration of PTSD with implications for the COVID-19 pandemic in association with a PubMed review conducted at the end of November 2020, finding 25 studies reporting PTSD related to COVID-19 in 26 published articles.

To present a carefully considered foundation for the discussion of this literature, the North et al. article included a detailed discussion of *DSM-5* criteria for PTSD to inform on the application of the PTSD criteria to these COVID-19 studies. The consideration of PTSD criteria was divided into three main elements that are essential for understanding trauma-related psychopathology: objectively defined trauma, objectively defined exposure, and subjectively reported reactions that must be conceptualized and considered separately from one another to think clearly about trauma-related psychopathology and its application to COVID-19. Starting with the definition of trauma in the PTSD criteria, a major impediment is quickly encountered: naturally occurring illness, such as in a COVID-19 pandemic, does not constitute a qualifying trauma for the *DSM-5* definition of PTSD. If the SARS-CoV-2 virus is not classified as a trauma agent, then exposure to a qualifying trauma is not possible with COVID-19 and any symptoms related to this illness cannot be considered “posttraumatic” given that COVID-19 is not classified as trauma. The article then concluded that PTSD is not a viable mental health outcome of COVID-19 given the current criteria. The symptoms related to COVID-19 exposure or infection must therefore likely represent manifestations of other established disorders or stressor-related distress symptoms or syndromes. The North et al. article further noted that none of the studies in their review of articles on COVID-19 and PTSD addressed the restriction in the criteria that naturally occurring medical illness as exemplified by COVID-19 cannot be considered trauma on which the diagnosis of PTSD depends.

An exemplary COVID-19 article included in this Special Issue is a study by Guo et al. [10] of symptoms of depression and psychological distress in a convenience sample of 2130 population members in China. Despite the study’s reliance on brief nondiagnostic online self-report symptom questionnaires, the psychological constructs measured were appropriate for the context of a pandemic without attempting to measure trauma-related conditions such as posttraumatic stress.

Not unexpectedly, this study found that the presence of higher numbers of COVID-19 cases in the surrounding community and personal social networks was associated with higher numbers of depression and distress symptoms and that greater contact with COVID-19 content in social media was associated with greater distress. However, the practice of social distancing as a public health and safety measure applied to the pandemic had mixed results, being associated with greater distress but with lower numbers of depressive symptoms. It is readily understandable that being surrounded by COVID-19 cases, being engulfed in COVID-19 communications, and having to practice social distancing might contribute to psychological distress. However, the authors noted that the finding of fewer depressive symptoms among those who practiced social distancing is consistent with other studies reporting less depression in those with high compliance with precautionary recommendations for preventing COVID-19 spread. Based on their findings, the authors suggested that public health approaches may help constrain pandemic-associated depression and distress first through provision of reliable information to overcome social media misinformation and second by encouraging the public to accept and embrace health practices to limit viral spread.

## 4. Conclusions

Contained within what might at first seem to be a loosely collected medley of disaster mental health research are valuable insights that resonate across the studies in this Special Issue. These articles were selected for their diversity in types of samples studied, mental health outcomes examined, and different methodological approaches to allow for the formulation of conclusions across these different approaches. The selection of articles was also driven by specific aspects of methodological rigor such as representative sampling and structured diagnostic interviews assessing the full criteria for psychiatric disorders. Given the array of material included in this Special Issue, a number of overarching comments and conclusions can be made.

A first and fundamental lesson in disaster research addressed in this collection of articles is that not all post-disaster mental health findings are necessarily specific to the disaster. Two important means of addressing the specific disaster relationship are to examine the findings in relation to the timing of the disaster and to compare disaster trauma-exposed and -unexposed samples. Two articles in this collection demonstrate the importance of timing of outcomes in relation to the disaster [2,5]. Because much or even most of the post-disaster mental health findings may have been present before the disaster and thus causally unrelated to the disaster, an analysis of pre-disaster along with post-disaster data permits the isolation of incident or new psychosocial sequelae of the disaster. In these two articles presenting both pre-disaster and post-disaster psychopathology, less than one-half of the post-disaster psychopathology represented incident psychopathology; furthermore, multivariate models demonstrated different predictors of post-disaster vs. incident psychopathology.

Two articles in this collection also compared disaster trauma-exposed and -unexposed samples [1,5], both demonstrating greater post-disaster psychopathology in exposed than in unexposed samples. Methodological rigor in these studies was demonstrated by the adherence to established criteria for trauma exposure for the diagnosis of PTSD, which is a pivotal issue for assessment of PTSD in disaster-exposed populations and necessitating differentiation of trauma from other types of stressors. A specific type of stressor examined in two studies in this review [1,4] was contact with disaster-related media, and both articles were adherent to the PTSD criteria definition of trauma exposure that specifically excludes witnessing or hearing of the disaster only through media.

A second major point to be appreciated from this collection of studies is that they examined an array of mental health outcomes, illustrating that even though PTSD is vital to disaster research, there are many other psychosocial aspects of disaster that are important to understand to inform on the full extent of disaster mental health needs and most effective responses to them. Besides PTSD, psychosocial aspects of disaster examined in these studies included depressive and anxiety symptoms and disorders, behavioral and substance use disorders, psychological distress, functional impairment, and not only negative outcomes but also positive outcomes such as posttraumatic growth and coping. A related third methodological feature of one of these studies [1] was the inclusion of qualitative data along with the more traditional quantitative data collection. Quantitative approaches using responses limited to structured fields with questions limited by the researchers’ awareness of issues and conceptual biases not only risk a tunnel-vision focus on PTSD but also may miss valuable insights voiced by survivors relating their stories by their own direction.

A fourth point worthy of comment is that difficulties comparing and contrasting findings from the published literature created by methodological inconsistencies across studies are practically the rule. Two articles in this Special Issue [2,3] demonstrated that this problem can be addressed by studying a series of disasters with consistent methodology so that the data can be combined into a single database for comprehensive analysis. This research strategy also demonstrated that the heavy research burden of collecting highly advantageous structured diagnostic interview data limiting sample sizes in individual studies can be overcome by combining structured diagnostic interview data from separate smaller disaster samples. A fifth and final point, relevant to an article in this Special Issue [5] is that, although older data are often undervalued, older data collected with rigorous research methods may provide important new knowledge not previously available from existing studies (and perhaps unlikely to be provided by future studies). This article illustrates that older data should not necessarily be rejected based on age alone but considered based on scientific merit.

Finally, the findings from this collection of articles have implications for disaster mental health interventions. Perhaps most importantly, the results of several of the studies in this Special Issue suggest that approaches to disaster-related experiences not qualifying as traumatic exposures cannot be neatly incorporated into established PTSD treatments. This is of particular relevance to the current COVID pandemic, as the North et al. review article makes the nosologic (and highly treatment-relevant) point that the experience of the COVID-19 illness itself and the social stressors in general populations brought on by the pandemic cannot be considered substrates for PTSD, thus warranting interventions for distress rather than PTSD-specific interventions. Consideration is also needed for evidence-based treatment of post-disaster disorders other than PTSD that may be present (e.g., MDD) as well as interventions for disaster-related distress not rising to the level of psychopathology.

As illustrated in this Special Issue, disaster research and its methodology are complex, but they also have direct treatment implications. This editorial highlighted some of these complexities in this Special Issue’s collection of articles. These complexities in disaster research have not received sufficient prior attention and depth of consideration in the existing literature. This discussion provides the disaster mental health research field with a glimpse of several specific problematic disaster mental health research issues with recommendations for circumventing them and conducting further research to resolve them.
